# Pulmonary Involvement in Microscopic Polyangiitis: Computed Tomography Findings in 55 Patients With Analysis of Risk Factors for Recurrence

**DOI:** 10.7759/cureus.21285

**Published:** 2022-01-16

**Authors:** Takeshi Saraya, Yukari Ogawa, Keitaro Nakamoto, Masachika Fujiwara, Haruyuki Ishii

**Affiliations:** 1 Respiratory Medicine, Kyorin University School of Medicine, Mitaka, JPN; 2 Pathology, Kyorin University School of Medicine, Mitaka, JPN

**Keywords:** lung, thoracic computed tomography, radiological findings, recurrence, microscopic polyangiitis

## Abstract

Background and objective

Pulmonary involvement is seen in up to 30% of microscopic polyangiitis (MPA) patients. Pulmonary radiological findings for MPA have been scarcely reported to date. This study was conducted to evaluate computed tomography (CT) and clinical findings at the time of MPA diagnosis as predictors for systemic or lung recurrence.

Methods

We retrospectively reviewed the medical records and radiological data of 55 MPA patients with pulmonary involvement who were admitted to our hospital between April 2008 and December 2016.

Results

Aside from pulmonary lesions, lesions were found in the kidneys (52.7%), skin (7.3 %), and peripheral nerves (3.6%). Biopsies were performed for 29.1% of the patients, with an overall diagnostic accuracy of 78.9%. Parenchymal opacities (74.5%, mainly ground-glass opacities and reticular shadowing) were more commonly seen than airway abnormalities were (40.0%, mainly bronchiectasis). Systemic recurrence in the first year after diagnosis was found in 10.9% of the patients, and it mainly involved the kidneys or lungs. A serum WBC count ≥ 10,900/μL was a risk factor for predicting systemic recurrence within the first year after diagnosis according to the Cox regression analysis (HR 11.1, 95%CI: 1.3-95.9, p=0.028). Lung recurrence within five years after the diagnosis was observed in 9.1% of the patients. The incidences of reticular shadowing and honeycombing in thoracic CT at diagnosis were significantly higher in recurrence-positive patients than in recurrence-negative patients, but these differences could not be used to predict lung recurrence.

Conclusions

Ground glass opacities, reticular shadowing, and bronchiectasis are prominent thoracic CT findings for MPA. There are no radiological patterns capable of predicting recurrence. However, a serum WBC count ≥ 10,900/μL at diagnosis might be a predictive factor for systemic recurrence within the year.

## Introduction

Microscopic polyangiitis (MPA) is a systemic necrotizing vasculitis that affects small-caliber blood vessels, sometimes including those of the lungs. Previous studies have reported the frequency of lung involvement at 22%-29% [1] To the best of our knowledge, the clinical and radiological features of MPA have not yet been clearly described, and the risk factors for systemic or lung recurrences of MPA are unknown. This study used clinical observations and radiological findings derived from thoracic computed tomography (CT) scans to investigate whether data available at the time of diagnosis of MPA can be used to predict the likelihood of recurrence.

## Materials and methods

Patients and study design

We retrospectively reviewed the medical records of MPA patients admitted to our hospital, a regional referral center, between April 2008 and December 2016.

**Figure 1:**
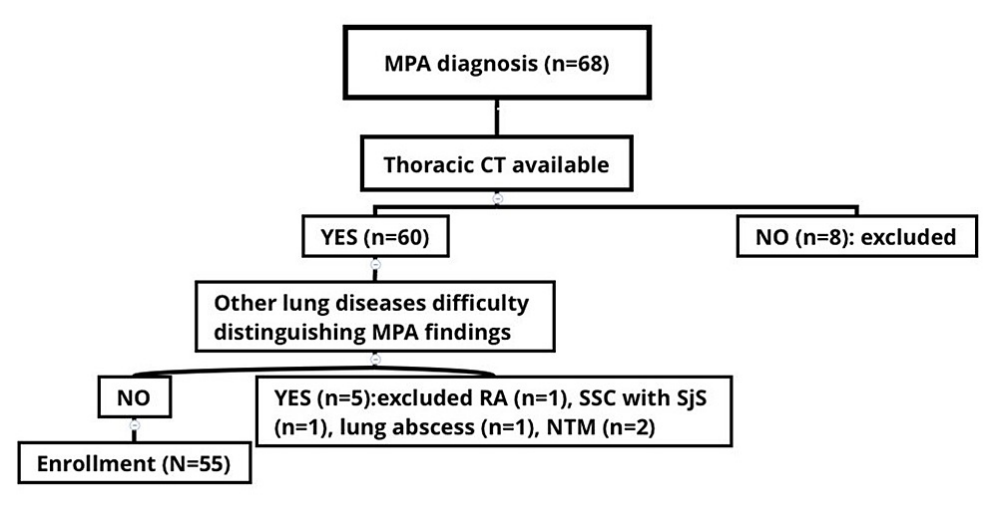
Schema for enrolling MPA patients Schema for enrolling MPA patients. Of 68 MPA patients, CT findings were available for 60. Five patients were excluded due to lung infections (nontuberculous mycobacterium [NTM], lung abscess) or collagen vascular diseases (Sjogren syndrome [SjS]  plus Scleroderma [SSC], Rheumatoid arthritis [RA]).

We enrolled 55 patients with lung involvement who satisfied the criteria for MPA given in Japanese Labour and Welfare, Japan 1998 but excluding patients who had no lung lesions detectable in thoracic CT scans, or for whom CT scans were not available (n=8), or who in addition to MPA had other lung conditions (n=5), such as lung infections (nontuberculous mycobacterium, lung abscesses) or collagen-related vascular diseases (Sjogren syndrome plus Scleroderma, Rheumatoid arthritis) (Figure 1).

**Figure 2:**
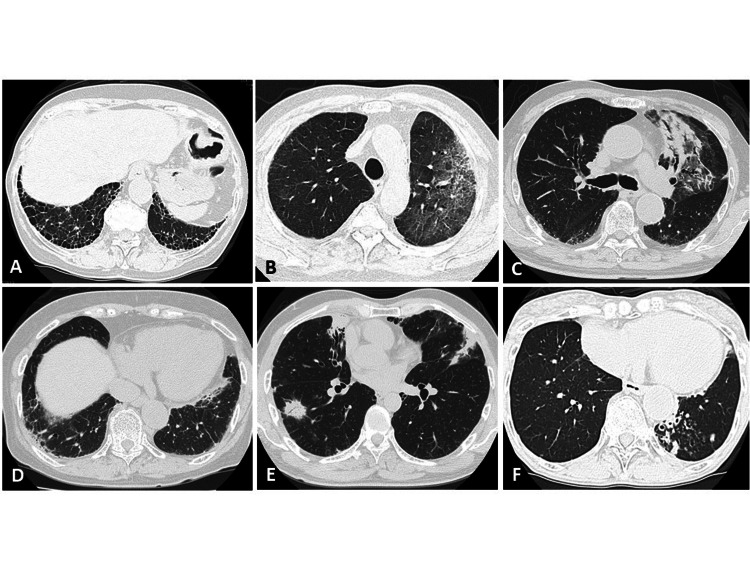
Representative radiological finding on thoracic CT Representative thoracic computed tomography findings for MPA, (A) showing honeycombing, (B) ground-glass opacities, (C) consolidation, (D) reticular shadowing, (E) nodules, and (F) bronchiectasis with centrilobular nodules.

The clinical and thoracic CT findings were evaluated at the time of diagnosis. Three pulmonologists, each with more than 10 years of experience, independently reviewed the high-resolution CT findings; decisions were interpreted by consensus. The thoracic CT findings were divided into two general categories: 1) airway abnormalities (bronchial wall thickening, bronchiectasis, centrilobular nodules); 2) parenchymal opacities (ground-glass opacities [GGO], consolidation, reticular shadowing, nodules, cavities, hemorrhage, honeycombing). Figure 2 illustrates representative thoracic CT findings.

Comparison of patients with and without systemic recurrence in the year following diagnosis

We compared clinical and radiological findings for the two groups defined according to whether there was systemic recurrence in the first year after the initial MPA diagnosis. Systemic recurrence was defined as the occurrence of one or more of the following events: 1) a newly observed increase in myeloperoxidase-antineutrophil cytoplasmic antibody (MPO-ANCA) titer, 2) progression of previously observed organ dysfunction, and 3) emergence of involvement of other organs.

Comparison of patients with and without lung recurrence in the five-year period following diagnosis

We also compared clinical and radiological findings for groups defined according to whether there was recurrence in the lungs in the five-year period after the diagnosis of MPA. We evaluated the findings available at the time of MPA diagnosis as predictive factors for recurrence in the lungs.

Statistical analysis

The data were analyzed statistically using a Pearson’s chi-squared test or Mann-Whitney U test. Statistical Package for the Social Sciences (SPSS) Statistics (IBM Corp. Released 2011. IBM SPSS Statistics for Windows, Version 20.0. Armonk, NY: IBM Corp.) was used for all analyses. The overall survival and recurrence rates were evaluated using the Kaplan-Meier method. A log-rank test was used to statistically compare the curves and p values. The cut-off point for the serum markers was determined as the minimum value based on the formula of [(1-sensitivity)2+(1-specificity)2]. A p-value < 0.05 in paired two-sided tests was considered significant. This study was approved by the ethics committee of Kyorin University (approval number 889 H28-149, dated Feb 10, 2017).

## Results

We identified a total number of 68 MPA patients over the study period. Eight were excluded owing to the absence of thoracic CT scans, and another five were excluded owing to complications of lung involvement by co-existing collagen vascular diseases such as rheumatoid arthritis (n=1), systemic scleroderma combined with Sjögren syndrome (n=1), lung abscesses (n=1), or non-tuberculous mycobacterium infections (n=2). The remaining 55 patients were enrolled in this study (Figure 1).

Patients’ clinical backgrounds

The median age of the 55 patients was 76 years (interquartile range [IQR]: 70-82 years), with a male-to-female ratio of 23:32 (Table 1). Among these 55 patients, 25 (45.5%) had respiratory diseases such as interstitial pneumonia (n=10), emphysema (n=6), old pulmonary tuberculosis (n=4), asthma (n=3), organizing pneumonia (n=1), or lung cancer (n=1). The organs affected at the time of diagnosis were the lungs (n=55, 100%), kidneys (n=29, 52.7%); skin (n=4, 7.3%), and peripheral nerves (n=2, 3.6%) (Table 1). It should be noted that out of the original set of 68 MPA patients, only 80.9% (n=55) showed lung involvement. The other eight, because they showed no pulmonary effects, did not undergo thoracic CT scans and were, therefore, excluded from this study.

**Table 1:** MPA patient clinical data CRP: C-reactive protein, KL-6: Krebs von den Lungen-6, LDH: lactate dehydrogenase, MPO-ANCA:  myeloperoxidase-antineutrophil cytoplasmic antibody, WBC: white blood cell count

Total number of patients	55
Age	76 (70-82)
Male/Female	23/32
Comorbid respiratory disease	25 (45.5%)
Interstitial pneumonia	10
Emphysema	6
Old pulmonary tuberculosis	4
Asthma	3
Organizing pneumonia	1
Lung cancer	1
Laboratory data	
WBC (x 10^3^/μL)	9,800 (7,300-13,000)
Hemoglobin (g/dL)	9.4 (7.7-10.9)
LDH (IU/L)	194 (168-237)
Albumin (g/dL)	2.6 (2.1-3.2)
CRP (mg/dL)	6.5 (1.0-11.9)
Creatinine (mg/dL)	1.2 (0.7-3.6)
MPO-ANCA (U/mL)	123 (62-296)
KL-6 (U/mL)	337 (243-539)
Involved organs	
Kidney	29 (52.7%)
Lung	55 (100%)
Peripheral nerve	2 (3.6%)
Skin	4 (7.3%)
Number of pathological assessments	16 (29.1 %)
Renal	13 (23.6 %)
Skin	4 (7.3 %)
Peripheral nerve	1 (1.8 %)
Lung	1 (1.8 %)
Diagnostic accuracy of biopsied specimens	15/19 (78.9%)
Renal	12/13 (92.3%)
Skin	2/4 (50%)
Peripheral nerve	1/1 (100%)
Lung	0/1 (0%)
Treatment	
Steroid pulse therapy	27 (49.1 %)
Prednisolone	53 (96.4 %)
Other immunosuppressants	14 (25.5 %)
Intravenous cyclophosphamide	9 (16.4%)

Tissue biopsies of the kidneys (n=13, 29.1%), skin (n=4, 23.6%); peripheral nerves (n=1, 1.8%), or lungs (n=1, 1.8%) were performed for some of the patients. The overall average diagnostic accuracy of biopsies was 78.9%. This can be decomposed into accuracies of 92.3% for renal biopsies, 50% for skin biopsies, 100% for peripheral nerve biopsies, and 0% for lung biopsies (Table 1). Serum inflammatory markers such as white blood cell counts (WBC) (median 9,800/μL, IQR: 7,300-13,000) and C-reactive protein levels (median 6.5 g/dL, IQR: 1.0-11.9) showed a mild to moderate increase from normal levels, and elevation of serum levels of the protein Krebs von den Lungen-6 (KL-6) (median 337 IU/L, IQR: 243-539) was also seen (Table 1). Approximately half of the patients (n=27, 49.1%) were treated using steroid pulse therapy.

Radiological findings at the time of diagnosis

Parenchymal opacities were more common (n=41, 74.5%) than airway abnormalities (n=22, 40.0%) in the CT findings of the 55 patients (Table 2). The observed parenchymal opacities comprised ground-glass opacities (n=22), reticular shadowing (n=18), consolidation (n=12), honeycombing (n=11), and nodules (n=9). The most frequently observed types of airway abnormalities were bronchiectasis (n=21) and centrilobular nodules (n=2) (Table 2). The radiological findings were comparable with or without systemic recurrence in the first year regarding airway abnormalities (16.7% vs 42.9%, p=0.38) and parenchymal opacification (100% vs 71.4%, p=0.32).

**Table 2:** Radiological findings at the time of diagnosis

Airway abnormalities	22 (40.0 %)
Bronchiectasis	21
Centrilobular nodules	2
Parenchymal opacification	41 (74.5 %)
Ground glass opacities	22
Reticular shadowing	18
Consolidation	12
Honeycombing	11
Nodule	9
Hemorrhage	7
Cavity	1
Pleural effusion	14 (25.5 %)
Pneumomediastinum	2 (3.6 %)

Clinical findings for patients with and without systemic recurrence in the first year

We found that systemic recurrence occurred within the year after diagnosis in six of the 55 MPA patients (10.9%). Systemic recurrences occurred in the kidneys (n=6), lungs (n=5), peripheral nerves (n=1), and central nervous system (presenting as hypertrophic pachymeningitis; n=1). The clinical characteristics and laboratory data at the time of diagnosis were similar for patients with and without recurrence, except for white blood cell (WBC) counts (Table 3).

**Table 3:** Clinical findings for patients with and without systemic recurrence in the year following diagnosis Data are expressed as median (interquartile range), CRP: C-reactive protein, CNS: central nervous system, KL-6: Krebs von den Lungen-6, LDH: lactate dehydrogenase, MPO-ANCA: myeloperoxidase-antineutrophil cytoplasmic antibody, WBC: white blood cell count *Organ involvement means deterioration of the organ dysfunction recognized at the time of diagnosis, or emergence of involvement of other organs.

	Systemic recurrence negative (n=49)	Systemic recurrence positive (n=6)	p value
Age	73 (62-77)	76 (70-81)	0.645
M/F	20:29	3:3	0.686
Comorbid respiratory disease (%)	22 (44.9%)	4 (66.7)	0.406
Laboratory data			
WBC (x 10^3^/μL)	9,100 (7,400-9,300)	14,350 (10,725-17,475)	0.022*
Hemoglobin (g/dL)	7.7 (7.1-9.7)	10.3 (9.4-11.7)	0.199
LDH (IU/L)	209 (160-258)	189 (178-249)	0.925
Albumin (g/dL)	2.3 (1.8-3.2)	2.8 (2.0-3.2)	0.945
CRP (mg/dL)	4.8 (1.4-9.8)	12.4 (2.2-16.9)	0.148
Creatinine (mg/dL)	0.8 (0.6-5.8)	2.3 (0.8-6.7)	0.365
MPO-ANCA (U/mL)	396 (67.5-511)	194 (81.5-545)	0.421
KL-6 (U/mL)	805 (693-1705)	384 (199-556)	0.896
Diagnosis of recurrence			
Re-elevation of titer of the MPO-ANCA	0	3	
Organ involvement*			
Kidney	0	1	
Peripheral nerve	0	0	
Lung	0	3	
IP		2	
Alveolar hemorrhage		1	
CNS	0	1	

**Figure 3:**
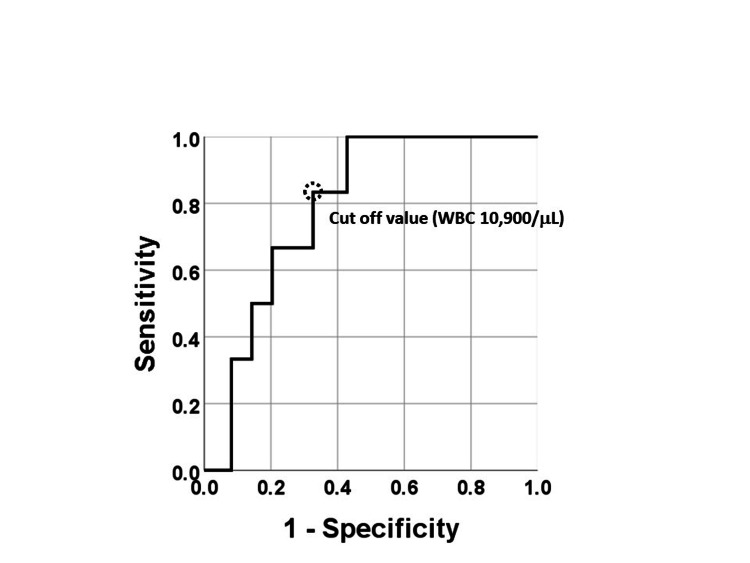
ROC curve for discrimination between systemic recurrence positive and negative groups in the year by using serum WBC count at the timing of initial diagnosis Receiver operating curve (ROC) for discrimination between groups with or without systemic recurrence within one year after diagnosis using serum WBC count at the timing of diagnosis

Between the systemic recurrence positive and negative groups, the most preferable threshold of WBC count for distinguishing between the two groups was 10,900/μL (sensitivity 83.3%, specificity 67.3%, area under the curve 0.753, 95%CI 0.559-0.948, p=0.044) (Figure 3). Based on the Kaplan-Meier method, the recurrence rate within the first year was significantly higher in the WBC count >10,900/μL group (n=20) than in the WBC count <10,900/μL group (n=31) (p=0.006). Furthermore, a WBC count >10,900/μL was a risk factor for predicting systemic recurrence within the first year according to the Cox regression analysis (HR 11.1, 95%CI: 1.3-95.9, p=0.028). However, the overall survival after a year between those two groups were comparable according to the Kaplan-Meier analysis (p=0.726).

Laboratory data at the time of diagnosis for patients with and without lung recurrence over five years

Lung recurrence occurred during the five-year period after diagnosis in 9.1% (n=5) of the patients, presenting as either acute exacerbation of interstitial pneumonia (n=4) or as alveolar hemorrhage (n=1). In laboratory tests performed at the time of diagnosis, the levels of serum MPO-ANCA (median 296, IQR:-79.0-401 U/m), WBC count (median 10050, IQR: 8,950-14,525 /μL), and KL-6 (median 663, IQR: 354-700 U/mL) appeared to be higher in the lung recurrence positive group than in the other patients ( [ MPO-ANCA, median 126, IQR: 44.5-233 U/mL] [WBC count, median 9800, IQR: 6,800-13,000/μL] [ KL-6, median 284, IQR: 238-492 U/mL]), but the difference was not statistically significant. Other clinical characteristics and serum laboratory data for the two groups were comparable. In the Cox regression analysis, the serum levels of MPO-ANCA and KL-6 at the time of diagnosis were not predictive of lung recurrence (MPO-ANCA; HR 1.0, 95%CI: 0.997-1.003, p=0.914) (KL-6; HR 1.0, 95%CI: 0.998-1.001, p=0.708) during the five-year follow-up period.

Radiological findings at the time of diagnosis for patients with and without lung recurrence within five years

The proportions of patients showing airway abnormalities or parenchymal opacification at the time of diagnosis were similar between the groups who did or did not show lung recurrence in the following five-year period (Table 4). The lung recurrence positive group, however, showed significantly higher incidences of reticular shadowing and honeycombing than the other patients did (reticular shadowing: n=4, 80% vs. n=14, 28%, p=0.035) (honeycombing; n=3, 60% vs. n=8, 16%, p=0.049). Honeycombing and reticular shadowing in the CT scans of lung recurrence positive group either progressed or was newly developed in the five-year period. However, Cox regression analysis could not predict lung recurrence in the five-year period based on the presence of reticular shadowing (HR 0.335, 95%CI 0.034-3.339, p=0.351) or honeycombing (HR 0.515, 95% CI 0.062-4.246, p=0.537). 

**Table 4:** Radiological findings at the time of diagnosis for patients with and without lung recurrence in the following five years

	Lung recurrence negative (n=50)	Lung recurrence positive (n=5)	p value
Radiological findings			
Airway abnormalities	19 (38 %)	3 (60 %)	0.379
Bronchiectasis	18 (36 %)	3 (60 %)	0.359
Centrilobular nodules	2 (4 %)	0 (0 %)	1.0
Parenchymal opacification	36 (72 %)	5 (100 %)	0.314
Ground glass opacities	19 (38 %)	3 (60 %)	0.379
Reticular shadowing	14 (28 %)	4 (80 %)	0.035*
Consolidation	11 (22 %)	1 (20 %)	1.0
Nodule	9 (18 %)	0 (0 %)	0.578
Honeycombing	8 (16 %)	3 (60 %)	0.049*
Hemorrhage	6 (12 %)	1 (20 %)	0.508
Cavity	1 (2.0 %)	0 (0 %)	1.0
Pleural effusion	14 (28 %)	0 (0%)	0.314
Pneumomediastinum	1 (2.0 %)	1 (20 %)	0.175

Comparison of the overall survival with or without recurrence in any organ

Using the Kaplan-Meier method, survival rates between the two groups with or without recurrence in any organ did not differ in the following period: one year (p=0.169), two years (p=0.843), and five years (p=0.843) after diagnosis. Similarly, the one (p=0.508), 2 (p=0.374), and five years (p=0.346) survival rates were comparable between the lung recurrence positive and negative groups.

## Discussion

Owing to the rarity of MPA, few reports of radiological findings have been published [2, 3]. Therefore, the identification of radiological and laboratory findings available at the time of diagnosis that is predictive of recurrence has been difficult. The present study is unique as radiological findings could be analyzed and the frequency of recurrence up to five years after the diagnosis of MPA was examined, permitting analysis of the factors that are relevant to recurrence. The data supports the following conclusions:

Based on radiological observations, the most frequently observed types of parenchymal opacities were ground-glass opacities and reticular shadows, while the most frequently observed airway abnormality was bronchiectasis. The CT findings for MPA were diverse in this study, but not substantially different from those previously reported [2, 3].

 The incidence of systemic recurrence in the first year after diagnosis was 10.9% (n=6), and the kidneys and lungs were the most frequently involved organs. Previous reports have described that the presence of antineutrophil cytoplasmic antibody (ANCA) in serum was a better predictor of lung fibrosis than the presence of proteinase-3 (PR3)-ANCA was, but it did not affect the recurrence of ANCA-associated vasculitis. This was also seen in our study. Furthermore, no predictor has been shown to reliably guide therapeutic decision-making [4, 5]. Hatemi et al reported that an increase of PR3-ANCA titer may help to predict relapses in ANCA-associated vasculitis patients and PR3-ANCA may also be associated with better response to rituximab [6]. Similarly, Schirmer et al. [7] analyzed PR3-ANCA positive granulomatosis with polyangiitis (GPA), myeloperoxidase (MPO)-positive GPA, and MPO-ANCA positive MPA and found that increased relapse rate was observed among patients with PR3-ANCA irrespective diagnosis. While, this study demonstrated that patients with recurrence had significantly higher serum WBC counts than patients without recurrence did, and high WBC counts (≥ 10,900/μL) at the time of diagnosis might be a potential marker for predicting systemic recurrence within the first year. 

Lung recurrence occurred in 9.1% of MPA patients within five years of diagnosis. Although the incidence of reticular shadowing or honeycombing in thoracic CT scans at diagnosis was significantly higher in recurrence-positive patients than in the negative patients, these CT findings could not predict lung recurrence according to the Cox regression analysis. A previous report suggested that pulmonary interstitial fibrosis, including honeycombing, can be an early manifestation of MPA, preceding the onset of vasculitis by several years [3]. However, the present study clearly demonstrated that fibrosis by itself cannot predict lung recurrence within five years after diagnosis. The risk factors for lung recurrence are therefore numerous, implying that it will be difficult to predict lung recurrence based on only laboratory and radiological findings at diagnosis.

Furthermore, this study demonstrated that neither lung nor systemic recurrence involving any organ seemed to affect the overall survival; however, more index cases will be needed to confirm these results.

This study illustrates MPA as a systemic disease that most frequently involves the lungs and kidneys. Only 29.1% of patients were examined pathologically, but the diagnostic accuracy in these cases was 78.9%. This result should not be interpreted as an assurance of diagnosis based on tissue biopsy of any organ, because a lung biopsy showed only necrotizing vasculitis affecting small vessels including arterioles, venules, or capillaries, even in autopsied specimens, as previously reported [8]. This suggests that pulmonologists should avoid using lung biopsies to diagnose MPA.

This study has some limitations. First, it was a retrospective study involving a small number of MPA patients. Second, all the enrolled patients showed pulmonary involvement on thoracic CT, which constitutes a selection bias. However, this was a necessary price to pay in order to make use of information derived from thoracic radiological findings to predict systemic and lung recurrence.

However, this study demonstrated that thoracic involvement in MPA was primarily characterized by parenchymal opacities (n=40, 74.5%), such as ground-glass opacities and reticular shadowing, and to a lesser degree by airway abnormalities (n=22, 40.0%), mainly presenting as bronchiectasis. None of the laboratory or radiological findings available at the time of diagnosis could predict lung recurrence during the following five years. However, serum WBC count (≥ 10,900/μL) at the time of diagnosis could be a potential marker for predicting systemic recurrence within the first year.

## Conclusions

Thoracic involvement in MPA was primarily characterized by parenchymal opacities (ground-glass opacities and reticular shadowing) followed by airway abnormalities mainly presenting as bronchiectasis. Although none of the laboratory or radiological findings available at the time of diagnosis could predict lung recurrence during the following five years, serum WBC count (≥ 10,900/μL) could be a potential marker for predicting systemic recurrence within the first year.
